# The Science and Practice of Medicine

**Published:** 1866-12

**Authors:** 


*The Science and Practice of Medicine. By William Aitken, M. D., Edin., Professor of Pathology in the Army Medical School, etc. In Two Volumes. Lindsay* & *Blackiston, Philadelphia,* 1866.

We have received, from the publishers, the first volume of the above work, with a promise of the second, so soon as published.

